# Nutritional Deficiencies in Children with Celiac Disease Resulting from a Gluten-Free Diet: A Systematic Review

**DOI:** 10.3390/nu11071588

**Published:** 2019-07-13

**Authors:** Giovanni Di Nardo, Maria Pia Villa, Laura Conti, Giusy Ranucci, Claudia Pacchiarotti, Luigi Principessa, Umberto Raucci, Pasquale Parisi

**Affiliations:** 1 Chair of Pediatrics, School of Medicine and Psychology, NESMOS Department, Sapienza University of Rome, S. Andrea University Hospital, 00189 Rome, Italy; 2 Department of Medical-Surgical Sciences and Translational Medicine, Sant’Andrea Hospital, Sapienza University of Rome, 00189 Rome, Italy; 3 Pediatric Unit, AORN Santobono-Pausilipon, 80129 Naples, Italy; 4 Pediatric Emergency Department, Bambino Gesù Children Hospital, IRCCS, 00165 Rome, Italy

**Keywords:** celiac disease, gluten-free diet, gluten-free products, nutritional deficiencies

## Abstract

Background: A strictly gluten-free diet (GFD) is the basis for managing celiac disease (CD). Numerous studies have reported nutritional deficiencies/imbalances ascribable to a GFD. The aim of this review is to describe nutritional deficiencies observed in children with celiac disease on a GFD, to discuss the clinical consequences related to these nutritional imbalances, and to identify strategies that may be adopted to treat them. Methods: We reviewed the MEDLINE and EMBASE databases between January 1998 and January 2019. Results: Children are, regardless of whether they are on a gluten-free diet or not, at risk of consuming too much fat and insufficient fiber, iron, vitamin D, and calcium. These imbalances may be exacerbated when children are on a gluten-free diet. In particular, the intake of folate, magnesium, zinc, and foods with a high glycemic index in children with CD who are on a GFD is significantly altered. Conclusions: Therapeutic protocols should include nutritional education to help teach subjects affected by disorders such as CD the importance of labels, the choice of foods, and the combination of macro- and micronutrients. Children with CD on a GFD should be encouraged to rotate pseudo-cereals, consume gluten-free commercial products that have been fortified or enriched, and use foods that are local and naturally gluten-free.

## 1. Introduction

Celiac disease (CD) is a systemic, immune-mediated, enteropathic disorder that is triggered by gluten in the diet in genetically-predisposed individuals [1] and is due to a complex interaction between dietary, immunological, and environmental factors [2]. Gluten is a protein complex present in wheat, rye, and barley. Celiac disease is characterized by a wide range of clinical symptoms, a specific serum autoantibody response, and varying degrees of damage to the small intestinal mucosa [1,2].

The prevalence of CD in Western populations is estimated to be approximately 1%, though the true prevalence of CD is difficult to establish owing to differences in clinical presentations, with a certain proportion of patients having few or no symptoms [3]. The reported prevalence of CD has increased markedly in the last years following the widespread use of anti-endomysial and anti- transglutaminase antibodies, which has resulted in the recognition of non-classical-CD [4].

CD is characterized by malabsorption due to villous damage in the small intestine. This malabsorption results in a number of nutritional deficiencies that involve macro- and micronutrients [2,4]. A strict gluten-free diet (GFD) is the only treatment strategy available for CD in clinical practice. If a GFD is adhered to strictly, symptoms may resolve, histological and laboratory findings may return to normal, and the risk of CD-related complications may drop in the vast majority of patients [2]. Previous studies have shown that nutritional deficiencies affect 20%–38% of CD patients [5,6,7,8,9,10,11,12,13,14,15,16,17,18]. The nutritional deficiencies observed in celiac disease may be due to CD itself and/or be a consequence of the GFD. The severity of these deficiencies may be affected by the time elapsed before CD is diagnosed or treated, the location and extent of damage to the small bowel, as well as the degree of malabsorption [19]. Although GFD is associated with better health, in the last twenty years numerous studies have reported that a GFD may cause nutritional deficiencies and imbalances. In this regard, it should be borne in mind that any restricted diet may cause a nutritional imbalance in both macro- and micronutrients, including minerals [20]. As this imbalance may occur even in the presence of histologically-confirmed remission, it cannot be ascribed to the ongoing ingestion of gluten and the consequent malabsorption [5,6,7,8,9,10,11,12,13,14,15,16,17,18]. Furthermore, nutritional deficiency and coexisting autoimmunity may lead to neurological dysfunctions in CD [21]. The negative consequences of a GFD may be due to a number of factors, including an altered food intake distribution, the relatively low quality of commercially available gluten-free products, and dietary practices that favor certain groups of food [22]. 

The aim of this review is to describe nutritional deficiencies observed in children with celiac disease on a GFD, to discuss the clinical consequences related to these nutritional imbalances, and to identify strategies that may be adopted to treat them.

## 2. Methods

We reviewed the databases of MEDLINE and EMBASE using various combinations of the following search terms: celiac disease, children, gluten, gluten free, gluten-free diet, nutritional, nutritional deficiencies, macronutrient and micronutrient. Observation study, randomized clinical trial, meta-analysis, systematic review, and consensus conferences were included. Studies regarding gluten sensitivity were excluded. Papers with information on at least one nutrient of our interest were included.

Data extracted from each eligible study were specifically analyzed in terms of nutrition quality of the diet, in particular excess or deficiency of particular nutrients. All relevant studies that had been published in English since 1998 were considered for the purposes of this review. 

Of the 178 identified articles, we excluded 130 on the basis of the title and abstract as they did not fit with our interest. The full text of the remaining 48 articles was extensively reviewed to determine whether the articles met the inclusion or exclusion criteria. Thirteen articles did not meet our inclusion criteria. The remaining 35 articles were considered for the review. Any other articles identified by reviewing the reference lists of relevant articles were included if considered appropriate.

## 3. Body Composition in CD Patients on a GF Diet

The data available in the literature on body composition and anthropometric parameters in children with CD on a GFD are conflicting. The findings of some studies indicate that strict adherence to a GFD reduces fat, leads to the recovery of a slim body mass [23], and normalizes the body mass index (BMI) in both underweight and overweight subjects [24,25] in addition to accelerating linear growth [26]. In one study by Brambilla et al., the authors found that being overweight and obese was less common among children with CD, both at diagnosis and during a GFD, than among healthy controls [27]. In another study Alzaben showed that although children with CD had a higher glycemic index and glycemic load than controls, weight-for-age z score and height-for-age z score were not different between groups [15].

By contrast, the results of other studies have suggested that a GFD may exert a negative effect on a number of anthropometric parameters in CD subjects [7,16]. For example, Mariani et al. found that there was a high prevalence of being overweight and obese in adolescents with CD on a GFD. However, the use in their study of body weight >110%, as opposed to the BMI, as a means of defining ‘being overweight’ is likely to have led to an overestimation of the number of overweight adolescents [5]. In another study conducted on 149 children with CD, Valletta et al. showed that there was a significant rise in the BMI z-score associated with a GFD, with the proportion of overweight subjects almost doubling [28]. A more recent case-controlled study reported that there were significant differences in certain body composition parameters (i.e. fat mass, fat-free mass, muscle mass, total, intracellular and extracellular body water, and body cell mass) as well as nutritional indicators (body mass index and body cell mass index) between children affected by CD and healthy controls. Indeed, a trend was observed in subjects on a gluten-free diet towards elevated indices of certain body composition components, and in particular fat mass [29]. 

The possible reasons underlying the unwanted weight gain and obesity reported in the aforementioned studies are the increased intake in calories due to the improvement in intestinal mucosal absorption and to the increased consumption of less complex carbohydrates and of more saturated fats during a GFD. These contrasting data may, however, also in part be due to differences in the timing of the anthropometric evaluation. Indeed, when children with CD are placed on a GFD, they often gain an excessive amount of weight before exhibiting catch-up growth and weight normalization [30]. 

Although the effects of a GFD on a person’s body weight and BMI remain a matter of debate, it is recommended that pediatricians closely follow the nutritional intake after a child has been diagnosed with CD, focusing on children who are already overweight upon presentation. 

## 4. Macronutrients 

### 4.1. Fat

Ingredients that are lipid-rich are a technical addition to the manufacturing of gluten-free bakery products that cannot be avoided [31,32]. All the studies except one [33] showed that the total and saturated fat content in gluten-free products (GFPs), and in particular for gluten free breads, pasta and bakery products, is higher than in gluten-containing products [34]. In a Spanish study in which 17 gluten-free brands were compared with 16 brands of equivalent gluten-containing products, Miranda et al. found that gluten-free breads tended to contain twice as much total fat, which was predominantly saturated fat, as the gluten-containing products. A comparable lipid profile was detected in pasta and bakery products [35]. 

A number of studies have reported a significantly higher total fat intake in children affected by CD than in healthy controls [8,12,17,18]. Other studies conducted by groups in Europe and Canada have yielded similar results despite not reaching statistical significance [5,6,13,15]. Only in one Italian study on 18 children did the authors show that CD patients consumed a significantly lower amount of fat as a percentage of energy than controls [11]. Care should, however, be taken when interpreting the data from this Italian study for the following three reasons: the CD patients enrolled also had a significantly higher calorie intake than controls, the small sample size and the fact that the CD patients consumed more fat than the controls, although the difference was not reported to be significant.

Interestingly, a number of studies has reported that total fat intake both in CD and in healthy children exceeds the recommended doses [5,6,9]. As these data indicate that fat intake in the general population itself is already high, a gluten-free diet, and the use of GF-rendered foods in particular, can only exacerbate such an imbalance [36]. With regard to saturated/polyunsaturated fat intake, both children with CD and healthy children have been found to consume higher amounts of saturated fat and lower amounts of polyunsaturated fat than recommended, though few studies have reported significant differences between the two groups. This probably reflects a more general trend in the population in the intake of fat subtypes that is not linked to a gluten-free diet [32].

### 4.2. Fiber

The fiber content in the starches and/or refined flours used to make GF products is usually low. Indeed, production entails removing the outer layer of grains (which contains most of the fiber), thereby reducing the final fiber content of the product [30,37].

Several studies and guidelines in adults demonstrated that the intake of dietary fiber provided by a GFD is often lower than that provided by a normal diet [38,39]. By contrast, studies on pediatric populations have shown that children fail to meet nutritional fiber intake recommendations regardless of whether they are on a gluten-free and gluten-containing diet [7,9,12,14,18], Mariani and co-workers being the only authors who have reported a lower fiber consumption in adolescents with CD than in healthy subjects [5]. These findings point to a population trend that reflects the contemporary Western diet, which is characterized by the replacement of fiber-rich plant foods and whole-grains with refined, processed foods [40]. 

The suggestion that children on a GFD may not be taking adequate fiber intake results from observations indicating that gluten-free foods are often composed of low-fiber starches and refined flours [39]. The considerably lower fiber level in GF dietary patterns that include lower grain consumption is thus likely to hamper the prevention of non-communicable diseases such as obesity, type 2 diabetes, and cardiovascular disease [41].

Studies in the literature do not, however, support this hypothesis. This is likely due to the fact that the fiber content of gluten-free products developed recently, which are based on alternative gluten-free grains and pseudo-grains such as quinoa, buckwheat, and amaranth, is equivalent to that found in wheat bread and might improve this situation [39].

### 4.3. Carbohydrates

Since gluten inhibits small bowel starch hydrolysis, excluding it from the diet may raise the glycemic response to carbohydrates [32]. Previous studies have shown that the glycemic index in GFPs is frequently higher than in gluten-containing products [30,42]. The only two studies that have investigated the dietary glycemic index in children with CD both found that in such children it was significantly higher than in control subjects [11,15]. This aspect, which is highly relevant to children with CD and type 1 diabetes, deserves to be investigated further. 

## 5. Micronutrients

As is to be expected, patients affected by CD who are not treated have deficiencies in the intake of micronutrients, including iron, folic acid, vitamins A, B6, B12, D, E, and K, copper, and zinc, due to malabsorption. It is noteworthy that the suboptimal micronutrient level upon diagnosis in a subgroup of patients persisted after the start of a GFD and thus required supplementation during follow-up [43]. When these nutrient deficiencies affect some subgroups of treated CD patients, they may contribute to extra-intestinal clinical manifestations, including neurological complications (epilepsy, cerebellar ataxia, peripheral neuropathy, neuromyotonia, myelopathy, dementia), psychiatric symptoms (paraesthesia, anxiety, depression) or bone alterations (osteopenia, osteoporosis).

It is usually possible to supplement almost all the micronutrients in CD patients on a GFD [43]. GFPs contain markedly lower levels of vitamins D, E, and B12, iron, folate, magnesium, potassium, and sodium than gluten-containing food [44]. It has also been demonstrated that only 5% of GF breads contain all four mandatory fortification nutrients (calcium, iron, niacin, and thiamin), while 28% are fortified with calcium and iron alone. However, it is important to consider that not all countries mandate that foods be enriched. In some countries only wheat based products are required to be enriched or fortified and those regulations do not pertain to “dietary or special foods” such as the GF products. 

This lack of fortification may increase the risk of micronutrient deficiency in subjects with CD who are on what is apparently an adequate GFD. Supplementation in these cases helps to normalize almost all micronutrient levels in subjects on a GFD, the only exception being vitamin D, which remains suboptimal [43]. 

### 5.1. Vitamin B

The average daily micronutrient intake of male and female patients, particularly of vitamins B1, B2, B6, and B9 (folate), has been shown to be significantly lower in celiac patients on a GFD than in the general German population [39], an issue that has been studied in adults though not in children affected by CD. In particular, when the total plasma homocysteine value (tHcy) was evaluated, significantly higher levels of total plasma homocysteine were observed in CD patients than in the general population [45]. In one recent study, when the tHcy level was measured as a marker of B vitamin status and the subjects’ general well-being was assessed in two CD populations either on or not on a GFD (i.e. supplemented with folic acid, cyanocobalamin and pyridoxine vs. not supplemented), tHcy levels returned to normal and general well-being improved in the supplemented group [46]. This lower vitamin intake may also be due to certain gluten-free cereal products, as is suggested by the findings of some studies showing that these products contain lower amounts of folate than gluten-containing products [45]. 

These findings suggest that hyperhomocysteinemia, which has recently been identified in the literature as an independent risk factor for cardiovascular disease, is due to nutritional factors [47]. Whether these mechanisms are applicable to a younger population of patients with CD has not been investigated, though it may be hypothesized that every effort should be made to prevent folate insufficiencies in children with CD from progressing into adulthood [48,49]. Indeed, it has been suggested that folate levels should be measured, and supplemented if required, in subjects with folate deficiencies [46]. In this regard, quinoa and amaranth contain 78.1 mg/100 g and 102 mg/100 g, respectively, of folic acid, which is more than wheat contains (40 mg/100 g). In addition, these cereals are good sources of vitamins (riboflavin, vitamin C, and vitamin E) [50].

### 5.2. Vitamin D

Vitamin D is known to play an important role in bone health and in regulating the immune system. Low levels of bone mineral density (BMD) have been reported in children with CD [51]. The reasons for poor bone health in children with CD are likely to be due to a number of factors, including inadequate intake (vitamin D and K, calcium), malabsorption, inflammation, and other lifestyle variables (weight-bearing physical activity, medication use, exposure to sunlight) [10]. Unsurprisingly, bone metabolism disorders and decreased bone mineral density have been reported in many of these patients with vitamin D-deficient levels [52]. 

Bone mineralization plays a central role in CD patients. A GFD has been shown to yield a significant improvement in mineral density after one year [53]. Unfortunately, bone mineral density cannot always be normalized by excluding gluten alone and a GFD often results in imbalances in calcium and vitamin D levels [54]. Guidelines state that the total 25 (OH) vitamin D level allows vitamin D deficiency to be diagnosed and monitored in CD patients [55]. 

There is evidence suggesting that vitamin D supplementation during a GFD prevents further bone loss, improves osteomalacia-related symptoms, and normalizes calcium levels. The data available on this issue are, however, still unclear, as are those regarding a possible correlation between vitamin D status and BMD in the general population. The American College of Physicians’ recent guidelines on the treatment of BMD and osteoporosis conclude that data on the efficacy of vitamin D, even in association with calcium, as a means of reducing the risk of fractures are contrasting and the overall effect is uncertain [56].

Few studies exist on the benefits provided by vitamin D supplements in CD patients. One study conducted on children and adolescents with CD reported that a 2-year course of calcium (1 g/d) and vitamin D (400 U/d) supplementation improved the BMD, though it should be borne in mind that the control population was not sex- and age-matched [57]. A study on 54 children with CD diagnosed by means of a biopsy detected hyperparathyroidism in 53.7% of the patients, hypocalcaemia in 40.74%, and vitamin D deficiency in 35.18%. After treatment consisting of a GFD lasting a mean period of 6 months, the resolution of hyperparathyroidism and hypocalcaemia was observed in all the patients while vitamin D levels returned to normal in 23 of the 34 patients [58]. Potentially inappropriate uses of vitamin D are, however, increasing [59] even though there is no evidence pointing to any advantage of vitamin D supplementation in patients with CD over that obtained by means of a GFD alone.

### 5.3. Minerals

Clinical studies designed to investigate the nutritional status of subjects on a GFD have shown that the intake of the minerals Ca, Fe, Mg, and Zn is insufficient. Although the data available on the content of mineral in products is still surprisingly scarce despite the widespread popularity of GFDs [20,44], they do suggest that a GFD may cause persistent micronutrient deficiencies [45]. 

One of the deficiencies most likely to result from a GFD is an insufficient amount of Ca, Fe, Mg, and Zn, this being ascribable to a considerable extent to the low mineral levels in the majority of popular gluten-free raw materials. More than 1 in 10 patients with CD on a GFD have been found to suffer from a mineral deficiency: magnesium and calcium in both sexes, zinc in men, and iron in women [60]. Few studies have been conducted on minerals in GFDs and the information available on micronutrients in gluten-free foods is poor. A recent paper in which a database of Ca, Fe, Mg, and Zn values in grain gluten-free products available worldwide was presented highlighted the absence of data regarding the content of Mg and Zn in most GF products [20]. Data about sodium content in GF products are conflicting and not conclusive.

Iron is an indispensable micronutrient that may be lacking in established celiac disease or be the presenting clinical feature even when diarrhea or weight loss are absent. Impaired duodenal mucosal uptake of iron is usually evident because the surface absorptive area in the duodenum is reduced, largely because celiac disease is an immune-mediated disorder that mainly targets the proximal small intestine [60]. Anemia, which is reported to occur in 5%–40% of patients in the West and in more than 80% of patients in developing countries, is one of the most commonly reported extraintestinal manifestations of CD. The most common form of anemia in CD is iron deficiency anemia (IDA). However, anemia in CD has actually been shown to be multifactorial and to have a number of different etiologies, including mixed nutritional deficiency, anemia of chronic disease (ACD), and aplastic anemia [61]. It is noteworthy, however, that 93% of the patients with CD in our study had anemia and that IDA was the most common cause (81.5%) [62]. A correlation emerged between the severity of anemia and the severity of villous atrophy. Iron deficiency may thus be considered to be a major nutritional problem in CD patients. In this regard, the results of studies indicate that iron stores may take a relatively long time (more than 6 months) to return to normal after small intestinal mucosa has healed [62]. Moreover, although mandatory fortifications have been applied to wheat flour as a vehicle for iron on a worldwide scale, these regulations are not applied consistently to gluten-free flours. This means that the risk of iron deficiency, which is reduced in children on normal diets, is high in CD patients [37]. It is thus of utmost importance that CD patients be advised to consume foods that are rich in iron, such as fruits, vegetables, and red meat [37].

Iron supplements should only be initiated after intestinal healing has occurred if required [54]. The most common way to replenish iron stores is to prescribe oral supplements until hemoglobin and iron deposit values return to normal. Parenteral iron may, however, be justified in specific situations (such as low tolerance, poor compliance, ineffectiveness, or clinical emergency) [63].

Zinc deficiency may markedly impact protein synthesis, leading to growth arrest, altered cellular function and a modified immune response state [64]. Caruso et al. reported that zinc deficiency can normally be solved by following a strict GFD for 1 year, thereby obviating the need for long-term supplementation. Magnesium deficiency may instead persist despite adherence to a GFD, probably owing to the low magnesium concentrations present in gluten-free cereal products [54]. Patients on a GFD should thus be encouraged to take magnesium-enriched foods.

Copper plays an important role in cellular transporters, which are called cuproenzymes and act as cofactors for a range of important enzymes. Copper is indispensable for the correct functioning of various organs and metabolic processes, including hemoglobin synthesis, iron oxidation, neurotransmitter biosynthesis, cellular respiration, pigment formation, antioxidant defense peptide amidation and connective tissue formation [65]. Although the association between copper deficiency (a potentially life-threatening condition) and CD is believed to be rare, no data are available on its true incidence; however, the fact that copper deficiency has been found in some CD subjects suggests that it may be an underdiagnosed condition. This hematological disorder may be fully resolved by administering copper supplements together with a GFD [66]. Hypocupremia should be investigated in patients with proximal limb weakness, even in the absence of clinically overt enteropathy [67]. Copper replacement combined with gluten restriction is likely to lead to the complete resolution of symptoms in such patients. 

It has been suggested that mineral deficiency may be overcome and the nutritional quality of a GFD be improved by replacing the low-nutritional raw materials used in GFDs with pseudo-cereals with a high nutritional value or through mineral supplementation [68,69]

## 6. Conclusions

The findings of studies in the literature show that children are at risk of consuming excessive amounts of fat and insufficient amounts of fiber, iron, vitamin D, and calcium, regardless of whether they are on a GFD or not. These imbalances may, however, be exacerbated by a GFD. Indeed, the intake of folate, magnesium, zinc, and consumption of foods with a high glycemic index in children with CD on a GFD is reported to be significantly altered.

Whenever a diagnosis of CD is made in a subject, nutritional deficiencies should be investigated and corrected, if required, with appropriate supplementation until full mucosal healing has occurred. The patient’s nutritional status should be closely monitored by means of blood tests both upon initiation of treatment and at follow-up visits. International guidelines suggest that patients on a GFD with deficiencies should be tested on an annual basis. 

The only available treatment for CD patients is a lifelong strict GFD. In Europe, adherence to a GFD by children is about 50% and inadvertent gluten contaminations are common [70,71]. Following a balanced diet and improving the GFD adherence are goals that could be achieved by explaining GFD with the help of dietitian and/or pediatric gastroenterologist experienced in the care of children with CD. Furthermore, a strict and regular follow-up of children with CD is advocated to ensure dietary adherence and to evaluate nutritional adequacy of the diet. Although, a recent retrospective study conducted on a children cohort [72] shown that 1 in 6 CD patients did not receive any education on GFD and 9% of the cohort had no regular follow-up after CD diagnosis. 

Recent studies have shown the helpful role of GIPs test (gluten immunogenic peptides) in the assessment of GFD adherence. On the basis of the availability, positivity of GIPs test could help families to understand if accidental transgressions occurred [73,74].

The distribution of daily calorie intake for a healthy and balanced diet suited to CD children on a GFD does not differ from that recommended for the general population.

Naturally gluten free foods should be chosen over manufactured gluten free products since the former have a higher nutritional value as regards energy provision, lipid composition, and vitamin content. The most beneficial naturally GF foods are those that are rich in iron and folic acid, such as green vegetables, legumes, fish, and meat [30]. Every effort should be made during dietary counseling to encourage the consumption of local naturally GF foods in order to promote a diet that is not only more balanced but also economically advantageous. 

Pseudo-cereals (e.g. amaranth, quinoa, and buckwheat, along with other minor cereals) may be considered a good alternative to other more commonly used ingredients in GFPs. Besides being a good source of carbohydrates, protein, dietary fiber, vitamins, and polyunsaturated fatty acids, these grains contain amounts of fiber that are higher than those in other plant foods and cereals and roughly the same as those in wheat. Moreover, they are a relatively good source of protein, which in terms of quantity and quality is better in pseudo-cereals than in wheat. The lipid content of pseudo-cereals is also higher than in other plant foods and is characterized by a higher proportion of unsaturated fatty acids (in particular α-linolenic acid). Amaranth and quinoa may also be considered good sources of folic acid, riboflavin, vitamin C, and vitamin E. Lastly, pseudo-cereals provide a wider choice of foods for children with CD and tend to be less expensive than standard GFPs.

CD patients and their families should be taught to pay special attention to the labeling and chemical composition of GFPs that are commercially available. Moreover, CD patients should be encouraged to use GFPs that have been enriched/fortified with vitamins and/or minerals in order to prevent deficiencies associated with GFD. 

In conclusion, nutritional education, aimed at understanding the importance of labels, the choice of food and the combination of macro- and micronutrients, should be incorporated into the therapeutic algorithm [75]. Furthermore, this diet-related education should encourage patients to use local naturally gluten-free foods such as pseudo-cereals, green vegetables, legumes, and fish.

## 7. Practice Tips

As complying to a gluten-free diet is the therapeutic basis for individuals with gluten-related disorders, every effort must be made to provide concrete solutions for the daily lives of these individuals. Here are some suggestions that have emerged from the latest research.

1. Provide patients with the name and telephone number of any local support groups. Face- to- face help enhances compliance and feelings of empowerment and reduces feelings of isolation.

2. Provide educational materials to address the patients’ most urgent needs. The materials may need to be divided into survival skills (which of the foods are gluten-free, what foods to avoid and where to source the foods locally), day to day coping (reading labels, recipes, etc.) and longer-term coping strategies (eating out and travel).

3. Set aside some time during follow-up visits to inquire about the clients’ adjustment to the gluten-free diet and lifestyle.

4. Encourage members of the patient’s family to attend follow-up visits as this provides an opportunity to discuss lifestyle adjustments.

5. Encourage any patients that seem to be having difficulties with the diet and/or compliance to make the most of support groups, social workers, or family counseling [68].

## Abbreviations

CD	celiac disease
GFD	gluten-free diet
GFP	gluten-free product
BMD	body mineral density
IDA	iron deficiency anemia.
